# Extended-Spectrum Beta-Lactamase-Producing *Escherichia coli* Meningitis and Cerebral Abscess in a Neonate: Therapeutic Challenge

**DOI:** 10.1155/2019/6874192

**Published:** 2019-03-28

**Authors:** Surasak Puvabanditsin, Marianne Jacob, Maaz Jalil, Samhita Bhattarai, Qaiser Patel, Mehrin Sadiq, Rajeev Mehta

**Affiliations:** ^1^Associate Professor of Pediatrics, Department of Pediatrics, Rutgers Robert Wood Johnson Medical School, One Robert Wood Johnson Place, New Brunswick, New Jersey 08903, USA; ^2^Department of Pediatrics, Rutgers RWJ Medical School, New Brunswick, New Jersey 08903, USA; ^3^Robert Wood Johnson University Hospital, New Brunswick, NJ, USA

## Abstract

We report a case of a 12-day-old term neonate with extended-spectrum beta-lactamase (ESBL) producing *Escherichia coli (E. coli)* meningitis and cerebral abscess. The patient received a 7-day course of antibiotics just few days prior to the infection. The incidence of infections from ESBL-producing *E. coli* is increasingly emerging. Antimicrobial agents must be vigilantly utilized to prevent the new highly resistant bacteria.

## 1. Introduction

After the initiation of intrapartum antimicrobial prophylaxis for *Streptococcus agalactiae* in the early 2000s [1], an increased rate of *ampicillin* resistance was reported in *Escherichia coli* [2, 3]. However, most of the *E. coli* isolates remained susceptible to aminoglycosides and extended-spectrum cephalosporins (ESC). Over the past decades, ampicillin and gentamicin/ESC are the empirical antibiotics for early-onset infection in neonates the majority of the neonatal intensive care units (NICUs) in the United States. Currently, extended-spectrum *β*-lactamases (ESBLs) are becoming an increasingly important cause of resistance to aminoglycosides and ESC in *E. coli*. We report a term neonate who was infected with ESBL-producing *E. coli* after a week of empirical antibiotics therapy. The lessons from our case confirm the emergence of ESBL-producing *E. coli* is a major problem not only in adults but also in neonates. Additional vigilance and attempts to establish an early diagnosis by care providers along with prompt deliverance of effective treatment would result in a better outcome.

## 2. Case Report

A 2650 g preterm female was born at 39 weeks' gestation to a 30-year-old Asian-Indian primigravida by induced vaginal delivery because of prolonged rupture of membranes (PROM). Prenatal care was unremarkable except for PROM (120 hours). The mother-to-be was admitted for induction of labor 2 days prior to delivery. She developed a temperature of 101 Fahrenheit during labor and received 2 courses of ampicillin and gentamicin. Vacuum suction was applied twice to assist the delivery. The amniotic fluid was noted to be “meconium-stained.” Apgar scores were 3, 6, and 7 at 1, 5, and 10 minutes, respectively. Intermittent positive pressure ventilation was applied for 3 minutes after which spontaneous breathing was noted. Physical examination revealed a weight of 2650 g (5th centile), length 52 cm (75th centile), and head circumference 33 cm (25th centile). Cephalhematoma was noted at the occipital area secondary to vacuum extraction. The baby had respiratory distress and was admitted to intensive care unit (NICU). During the first few hours of life, the patient required 100% supplemental oxygen via nasal continuous positive airway pressure (CPAP). Umbilical arterial and venous catheterizations were performed. Arterial blood gas at 1 hour of age showed pH 7.18, pCO_2_ 28, pO_2_ 44, and base deficit −17 meq/dL. Blood culture was obtained, and high-dose ampicillin and gentamicin were begun. Complete blood count at 12 hours of age showed high band count with a ratio of immature and total neutrophils (I/T ratio) of 0.5. Chest X-ray was unremarkable, and the patient rapidly recovered from respiratory distress. Umbilical arterial and venous catheters were removed within 24 hours of life. The blood culture was negative, and the patient was discharged after receiving a 7-day course of intravenous (IV) antibiotics.

Three days after hospital discharge, the patient was noted to be lethargic and had a poor oral intake. She was referred from the pediatrician office to the emergency department, where blood culture and lumbar puncture (LP) were performed. Cerebrospinal fluid (CSF) was cloudy. Intravenous ampicillin and gentamicin were given within an hour after LP. Immediately after admission to the NICU, ceftazidime was added to the treatment. The patient was febrile upon NICU admission. The Gram stain of the CSF specimen revealed numerous Gram-negative rods. Within 24 hours, the blood and CSF cultures revealed a growth of Gram-negative bacilli.

**Figure 1:**
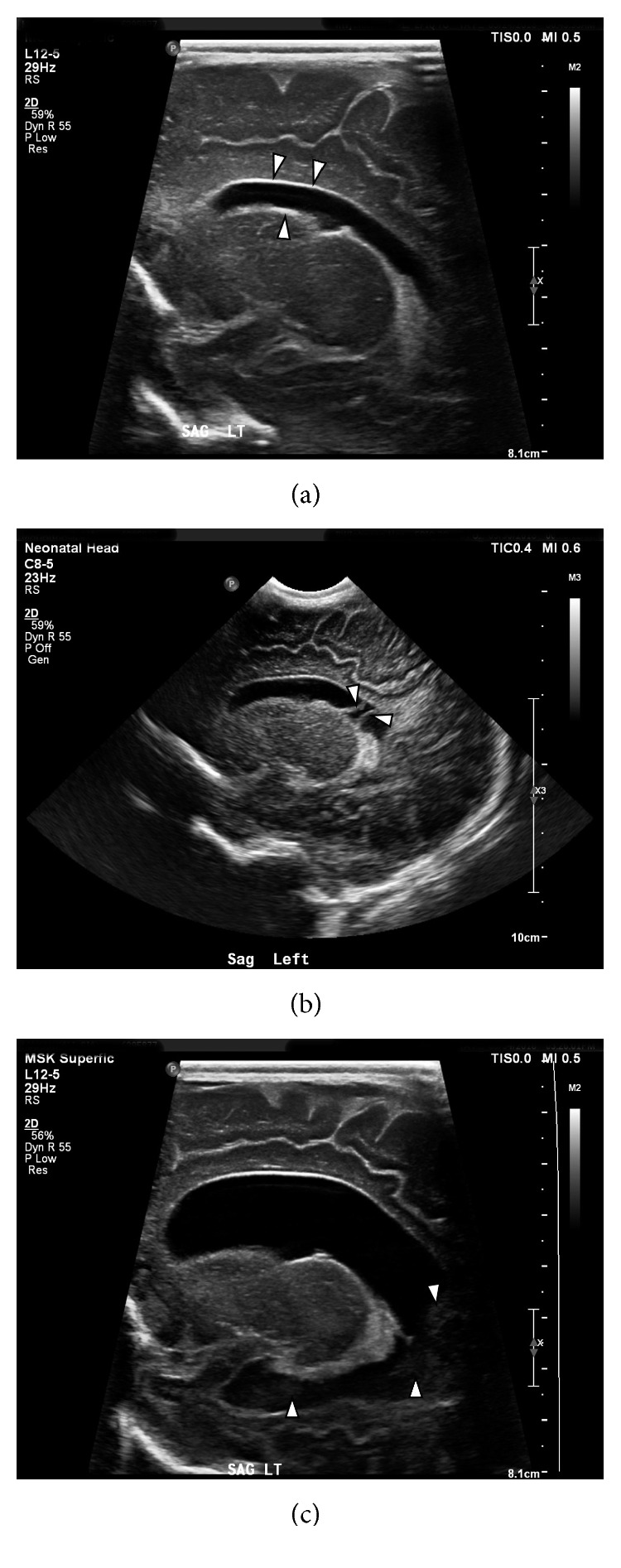
(a) Neurosonogram performed on the 2nd hospital day showed a mildly dilated left lateral ventricle and echogenic ependyma (arrows); findings associated with ventriculitis. (b) Hospital day 7: neurosonography showing mildly dilated left lateral ventricle, echogenic ependyma, and intraventricular debris (arrows), suggestive findings associated with ventriculitis. (c) Hospital day 11: neurosonography showing moderately dilated left lateral ventricle, echogenic ependyma, and intraventricular debris (arrows).

**Figure 2:**
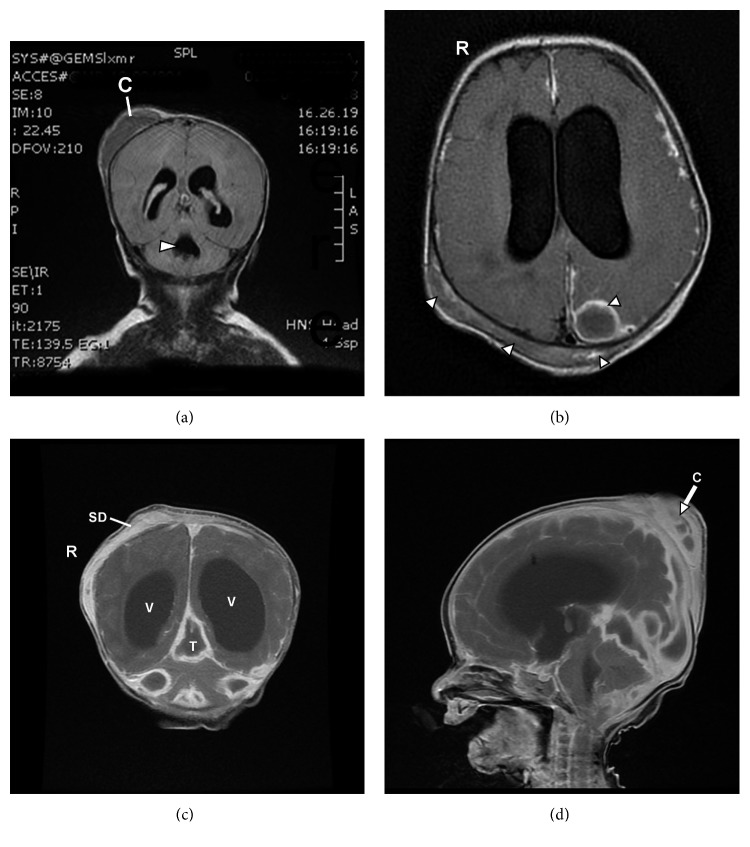
(a) MRI of the head performed on the second hospital day showing mildly dilated lateral and fourth (arrow) ventricles. Cephalhematoma (C) was also noted. (b) Hospital day 13: MRI of the head showing moderately dilated lateral ventricles. Bilateral subdural and left parietooccipital parafalcine fluid collections were noted, suggestive findings of multiple abscesses. (c, d) Hospital day 19: MRI of the head showing multiple enhancing lesions in the bilateral posterior fossa, tentorium (T), and subdural (SD) region, suggestive findings of multiple brain abscesses and subdural empyema. Lateral ventricles (V) were moderately dilated.

**Figure 3:**
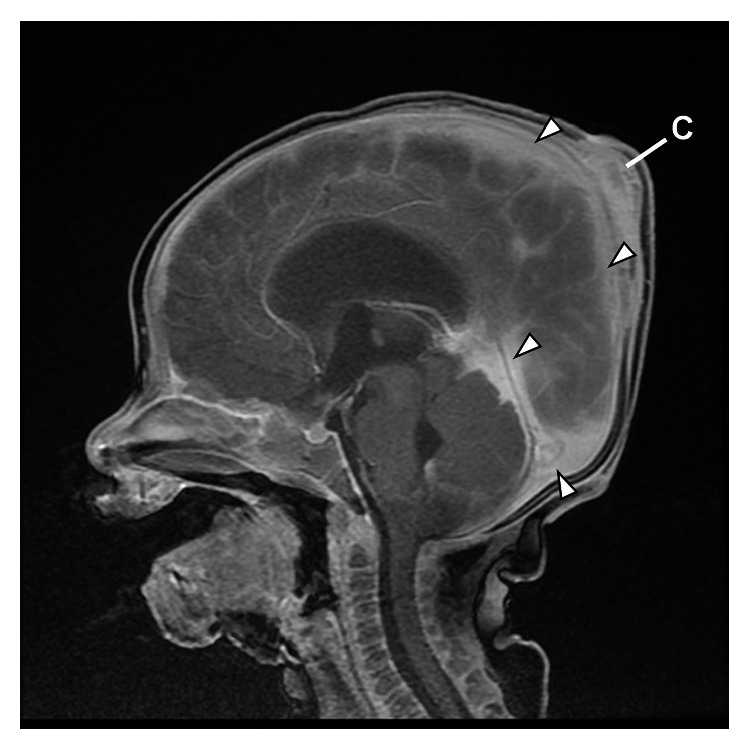
Hospital day 63: MRI of the head showing trace cerebral (falx and tentorium) and posterior fossa subdural collections (empyema) (arrows). C: cephalhematoma.

The bacteria were subsequently identified as *E. coli*. Seventy-two hours after admission, the initial broth microdilution antimicrobial susceptibility results (Microscan) for both isolates were as follows: resistant to ampicillin, gentamicin, tobramycin, cefazolin-ceftriaxone, levofloxacin, and sulfamethoxazole-trimethoprim; susceptible to meropenem, aztreonam, piperacillin-tazobactam, amikacin, and meropenem; and intermediately sensitive to ceftazidime and augmentin. Thereafter, IV meropenem and amikacin were begun. It took 5 days to sterilize the bacteremia, and the CSF became sterile 7 days after admission. Complete blood count showed an I/T ratio of 0.5. Serial MRIs were performed after 2 and 3 days, and 2, 3, 4, and 7 weeks of therapy showed subdural hydrocephalus, ventriculitis, abscesses, and subdural empyema (Figures 1 and 2). A ventricular tap was done 10 days after admission. Ventricular reservoir placement and subdural empyema tap were performed 14 days after admission. CSF culture from the ventricle was negative but the subdural exudate grew ESBL *E. coli*. She developed dysphagia after readmission to the NICU, and it took 11 weeks to achieve full oral feeding. After completion of 9 weeks course of meropenem and 6 weeks course of amikacin, she was discharged at 83 days of age; his weight was 4480 g (10th centile), length 58 cm (50th centile), and head circumference 38 cm (10th centile). Repeat MRI prior to discharge showed trace amount of the subdural empyema, mild ventriculitis, and ventriculomegaly (Figure 3).

## 3. Discussion

*Escherichia coli* are a part of the normal microflora of the vagina. Vertical transmission can occur from the mother to her newborn baby during the delivery process and may result in severe neonatal bacterial disease [4–6].

Predominance of Gram-negative bacteria in neonatal infections is attributed to the significant decline in the rate of group B streptococcal infection secondary to the universal screening at 35 or more weeks of gestation and intrapartum antibiotics for colonized women [1]. The gastrointestinal tract is the frequent reservoir of ESBL-producing *E. coli*, and reports have shown that transient carriage of ESBL-producing organisms on the hand of health-care workers or on artificial nails may also facilitate transmission [7, 8].

Studies have shown that the presence of infection due to ESBL-producing organism results in a high rate of inadequate treatment [9]. The patient with ESBL-producing organism is 9 times less likely to receive adequate antibiotic therapy 24 hours after blood culture was taken [10]. The negative impact of delayed initiation of adequate treatment is expected to be more pronounced in immune-incompetent patients such as neonates. ESBLs are often associated with plasmids and can carry genes that encode for coresistance to various antibiotics such as aminoglycoside, fluoroquinolones, and sulfamethoxazole-trimethoprim [11]. In our patient, the *E. coli* was resistant to ampicillin, gentamicin, tobramycin, cefazolin, ceftriaxone, sulfamethoxazole-trimethoprim, and fluoroquinolones. However, it was sensitive to amikacin. To this end, some authors have suggested rotating the gentamicin and amikacin for 6 months each, to minimize the emergence of resistance [11].

The aminoglycosides or cephalosporins are often a default choice for empirical treatment of neonatal infection in majority of NICUs in the United States. The association between colonization and subsequent infection with ESBL-producing organisms has been confirmed in multiple reports [9]. Therefore, screening high-risk patients such as high-risk newborn (e.g., prolonged rupture of membranes and prematurity) aiming at adjusting empirical treatment may be effective in improving the neonatal outcome.

The influence of different empirical therapies on patient outcome is difficult to assess. As soon as microbiological results reveal non-adequacy of the empirical regimen, it is changed to a targeted or definite treatment. The interval to this treatment adjustment varies according to availability of microbiological results. The reported clinical outcome of the treated patient is influenced by both, the empirical and the definite therapy. In our case, it took more than 72 hours before the definitive treatment was initiated. A recent retrospective multicenter study showed that insufficient empirical treatment was associated with increased mortality in patients with severe sepsis and shock [12]. Two studies showed some nonsignificant trends favoring imipenem and discarding cephalosporins as empirical therapy [13, 14].

The source of the resistant *E. coli* in this neonate is unknown. Review of the prenatal history did not reveal any risk factors for colonization or infection with an antibiotic-resistant organism. Although the mother had emigrated from India, she had not visited India for several years. She is a housewife, did not have any significant contact with patients, and had not received antibiotics during the course of the pregnancy except during the delivery. However, there was clinical evidence for chorioamnionitis. Nevertheless, the possibility of intrapartum acquisition secondary to the instrumentation (vacuum extraction) associated with the delivery cannot be excluded.

The association between colonization and subsequent infection with ESBL-producing *E. coli* has been confirmed in several studies. Hence, screening high-risk patients with the aim of adjusting the empirical treatment may be effective in improving patient outcomes. To further narrow the scope of the screening programs, important risk factors such as prior exposure to antibiotic treatment and healthcare contact could be applied. However, the approach of systematic screening and a consequential adaptation of empirical treatment have not been evaluated in a systematic fashion. Further infection control measures to cope with the emergence of ESBL-producing *E. coli* have been discussed. Unfortunately, results of studies regarding the impact of contact precautions, antibiotic restriction, and eradication of colonization are still inconclusive. More studies investigating the ways of transmission in ESBL-producing *E. coli* are needed to facilitate decisions on enforced contact precautions. Possible detrimental effects of contact isolation should also be taken into account.

In summary, in the high-risk setting such as immune-incompetent population (e.g., neonates), the presence of ESBL-producing organisms has negatively affected the clinical outcome by leading to increase rate of inadequate initial therapy and serious morbidity. Should carbapenems be favored to cephalosporins or aminoglycosides as empirical therapy in neonates? Should pregnant mother be screened if she prior exposed to antibiotic treatment and health-care contact? We are still lacking randomized trials or studies on these issues, especially in neonates.
